# Spinocerebellar Ataxia Type 3 Pathophysiology—Implications for Translational Research and Clinical Studies

**DOI:** 10.3390/ijms25073984

**Published:** 2024-04-03

**Authors:** Fabian Stahl, Bernd O. Evert, Xinyu Han, Peter Breuer, Ullrich Wüllner

**Affiliations:** 1 German Centre for Neurodegenerative Disease (DZNE), 53127 Bonn, Germany; fabian.stahl@dzne.de; 2 Departments of Neurology and Neurodegenerative Diseases, University of Bonn, 53127 Bonn, Germany; bernd.evert@ukbonn.de (B.O.E.); hxymed@yeah.net (X.H.); peter.breuer@ukbonn.de (P.B.)

**Keywords:** spinocerebellar ataxia 3 (SCA3), transcriptional and posttranscriptional regulation, ASO, miRNA, CRISPR/Cas, clinical trials, genomic modifier, compound modifier, high throughput screening, cholesterol, SREBP

## Abstract

The spinocerebellar ataxias (SCA) comprise a group of inherited neurodegenerative diseases. Machado–Joseph Disease (MJD) or spinocerebellar ataxia 3 (SCA3) is the most common autosomal dominant form, caused by the expansion of CAG repeats within the ataxin-3 (ATXN3) gene. This mutation results in the expression of an abnormal protein containing long polyglutamine (polyQ) stretches that confers a toxic gain of function and leads to misfolding and aggregation of ATXN3 in neurons. As a result of the neurodegenerative process, SCA3 patients are severely disabled and die prematurely. Several screening approaches, e.g., druggable genome-wide and drug library screenings have been performed, focussing on the reduction in stably overexpressed ATXN3(polyQ) protein and improvement in the resultant toxicity. Transgenic overexpression models of toxic ATXN3, however, missed potential modulators of endogenous ATXN3 regulation. In another approach to identify modifiers of endogenous *ATXN3* expression using a CRISPR/Cas9-modified SK-N-SH wild-type cell line with a *GFP*-*T2A*-*luciferase* (*LUC*) cassette under the control of the endogenous *ATXN3* promotor, four statins were identified as potential activators of expression. We here provide an overview of the high throughput screening approaches yet performed to find compounds or genomic modifiers of ATXN3(polyQ) toxicity in different SCA3 model organisms and cell lines to ameliorate and halt SCA3 progression in patients. Furthermore, the putative role of cholesterol in neurodegenerative diseases (NDDs) in general and SCA3 in particular is discussed.

## 1. Spinocerebellar Ataxia Type 3

Spinocerebellar ataxia type 3 (SCA3) or Machado–Joseph Disease (MJD) is an autosomal dominant inherited ataxia and the most common SCA worldwide, accounting for 20–50% of all SCA families [1]. The mean age of onset is between 30 to 40 years and progressive neurodegeneration over 10 to 15 years leads to premature death. SCA3 is caused by an expansion of the C-terminal polyglutamine (polyQ) tract in the ATXN3 protein. The length of normal polyQ alleles varies from 12 to 42 repeats whereas pathogenicity is induced by expanded alleles ranging from 60 to 87. The polyQ repeat length of SCA3 inversely correlates with the age of onset, misfolding, aggregation, and severity of symptoms [2]. 

Pathological hallmarks in SCA3 are widespread aggregation of ATXN3 protein and formation of inclusion bodies inside and outside of the cell nuclei leading to general loss of neurons [3,4].

## 2. Ataxin-3 Gene

The ataxin-3 gene (*ATXN3*) was first cloned by Kawaguchi and colleagues, who found an expanded polyQ coding CAG repeat in *ATXN3* of clinically diagnosed SCA3 patients [5]. The genomic locus of normal *ATXN3* comprises 11 exons, which encode the approximately 42 kDa large disease protein ataxin-3 (ATXN3) [6]. The CAG repeats were found to reside in exon 10 and confer when expanded, the neurotoxic properties of ATXN3 and increased molecular weight. As a result of alternative splicing, 20 different ATXN3 protein-coding mRNAs have been found [7]. In the brain, the most frequently expressed isoform consists of 11 exons [8].

## 3. Transcriptional and Epigenetic Regulation 

In contrast to ATXN3s, with various identified physiological roles, relatively little is known about the transcriptional regulation of *ATXN3*. Schmitt et al., 2003, characterised the *ATXN3* promotor and identified SP1 and CCAAT motif-binding factor (CBF) to bind the *ATXN3* promotor in vitro by using electric mobility shift assays (EMSA) [6]. Data for epigenetic modifications of *ATXN3* are scarce. DNA methylation levels of *ATXN3* promotors were analysed from SCA3 patients and controls. In the PBMC-derived DNA, hypermethylation of 7/17 CpG sites in the first CpG island has been found in SCA3 patients compared to controls. However, no correlation between *ATXN3* mRNA levels and hypermethylation was observed [9].

## 4. Posttranscriptional Regulation

It was found that *ATXN3* expression can be regulated post-transcriptionally via miRNAs. Different miRNAs like miR-181 and miR-25 family members but also miR-9 and miR-494 have been shown to directly bind to the 3′ untranslated region (3′UTR) of *ATXN3*. Luciferase assays with co-transfection of miRNA expressing vectors and *LUC* vectors, containing wild-type and mutated miRNA binding sites of the *ATXN3* 3′UTR sequence, were performed. The generated data revealed that the binding of miRNAs reduced the LUC signal for the plasmids with the wild-type sequence but not with the respective mutations. An insertion of the 3′UTR sequence of *ATXN3* in the coding sequence of a mutant *ATXN3* expressing plasmid and subsequent transfection into human embryonic kidney cells (HEK) led to a reduced expression of the protein and a decreased number of ATXN3 containing aggregates compared with the normal coding sequence. The same study showed that genetic silencing or pharmacological inhibition of Dicer and Drosha, key enzymes in miRNA biogenesis, leads to increased ATXN3 protein levels in HEK cells [10]. Expression levels of miRNAs were found to differ in SCA3 patients compared to controls. Whereas miR-25 and miR-181 family members are upregulated in SCA3 patient-derived lymphoblastoid cells, miR-9, miR-181a, and miR-494 have been shown to be decreased in human SCA3 brain samples and SCA3 models [11]. These differences may be due to different cell lines and samples. The regulatory importance of 3′UTR in *ATXN3* expression is further underscored by associations of new SNPs in the close distance to the conserved miR-25 binding site with early-onset SCA3 [12]. 

## 5. Structure and Function of ATXN3 Protein

**Figure 1:**
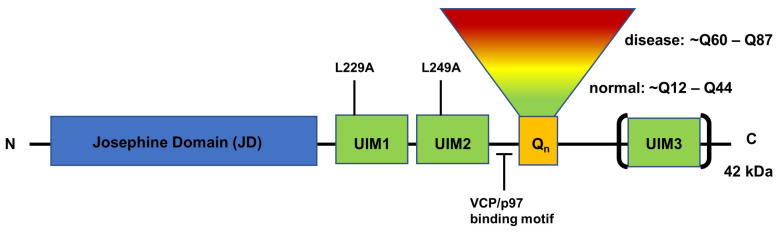
Protein structure of ATXN3. The N-terminal JD domain confers ATXN3 the DUB enzyme activity. The C-terminal region consists of two to three ubiquitin interacting motifs (UIM), dependent on the respective splice variant (brackets). Mutations in the conserved lysine of UIM1 and 2 cause differentiated binding affinity of ATXN3 to polyubiquitin chains. The binding of VCP/p97 is facilitated by an arginine/lysine motif upstream of the polyQ (Q_n_) expansion. A polyQ expansion from ~Q60 to Q87 causes onset of SCA3. Modified from Costa and Paulson, 2012 [18].

Structurally, the N-terminal domain of ATXN3 represents an evolutionary conserved Josephin domain (JD). The C-terminal harbours two neighbouring ubiquitin-interacting motifs (UIM) upstream and a third UIM downstream of the polyglutamine tract, dependent on the respective splice variant. Additionally, downstream of the JD, two nuclear export signals (NES) and a C-terminal binding motif for the valosin-containing protein (VCP) were reported [13,14,15]. More recently, nuclear magnetic resonance (NMR) studies of mixed-folded ATXN3 reveal new insights into its structural properties in solution and will help to understand the molecular recognition of polyubiquitin by its C-terminal UIMs (Figure 1) [16,17].

ATXN3 is ubiquitously expressed and detected in many different regions within the brain and was found to have a dendritic and axonal localisation in most neurons. Intracellularly, ATXN3 has mainly been detected in the cytoplasm but tiny amounts were also found in the nucleus [3,8,19]. Little is known about a putative role in glia cells or what mechanisms render neurons particularly vulnerable. 

Post-translational modifications like ubiquitination, were shown to increase the activity of ATXN3 but did not affect the translocation rate. However, it was observed that the enzymatically active protein was located more often in the nuclei [13,20].

**Figure 2:**
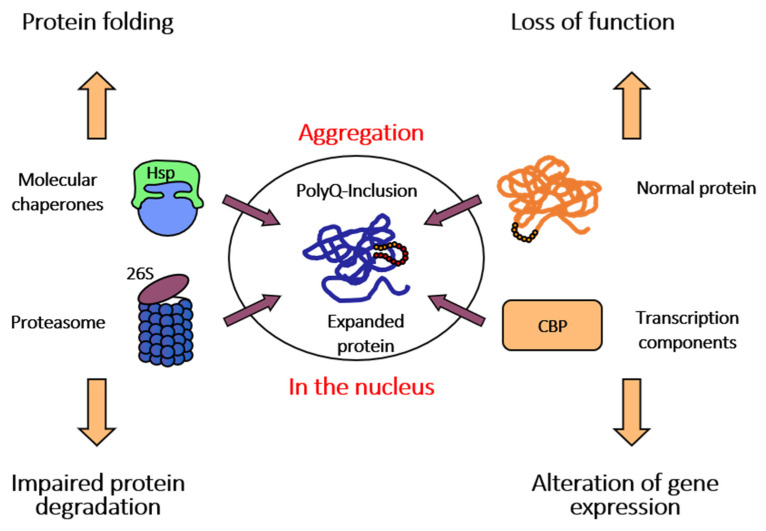
Multiple levels of polyQ toxicity in SCA3. Pathological expansion of the polyQ tract in ATXN3 affects several pathways within the cell, leading to neuronal demise and degeneration.

Despite the variety of ATXN3 cellular functions including participation in the ubiquitin–proteasome system (UPS), aggresome formation, cytoskeletal organisation, DNA binding, and interaction with transcription factors (TFs) it can likely be considered a non-essential protein since *ATXN3* knockout in mice or *C. elegans* did not show an obvious phenotype (Figure 2) [21,22].

According to the sequence homology to JDs and its UIMs, a potential function in the ubiquitin–proteasome system (UPS) of ATXN3 as a deubiquitinating enzyme (DUB) was assumed [23]. 

Indeed, it was shown that ATXN3 binds polyubiquitin chains with four or more ubiquitins, which are required for proteasomal recognition and degradation, with its two UIMs upstream of the polyQ stretch. Mutations of the conserved leucine (L229A) in the first UIM almost totally abolished the binding of polyubiquitin and similar mutations (L249A) in the second UIM inhibit binding to a lesser extent. Furthermore, the authors observed a potential ubiquitin–protease activity of ATXN3, which was reduced after applying a ubiquitin–protease inhibitor. Mutations in the UIM in wild-type ATXN3 did not affect the protease activity (Figure 1) [24]. 

Extended in vitro studies of the DUB properties of ATXN3 revealed a regulatory role of UIMs in the trimming of K48-linked ubiquitin chains. It was shown that ATXN3 cleaves K48-linked polyubiquitin chains to a length of 5–7 ubiquitin but at the same time inhibited the proteasomal degradation of the substrate. Mutations in the active site of ATXN3 (no protease activity) did not restore proteasomal degradation. Only mutations in the active site and of both C-terminal UIMs upstream of the polyQ repeat led to restored degradation indicating a critical role of UIMs for inhibiting proteasomal degradation. Reduction in proteasomal degradation is likely mediated by direct and continuous binding of UIMs to polyubiquitin chains of five to seven, which blocks the substrate from entering the proteasome. Mutations in both UIMs only, allow ATXN3 to also trim shorter ubiquitin chains, which potentially results in decreased substrate degradation [20,25]. Interestingly, a study from Rao et al. in 2020 showed that ATXN3s DUB activity can be dramatically activated by naturally occurring ubiquitin species [26].

Several ATXN3-regulated targets have been identified to play a role in the UPS. The ubiquitin E3 ligase parkin, a risk gene for familial PD, was shown to be deubiquitinated by ATXN3. Mutant ATXN3 polyQ led to a more effective de-ubiquitination and mediated subsequent degradation of parkin via the autophagy pathway. In addition, overexpression of mutant ATXN3 polyQ in mice showed reduced parkin protein levels in brain lysates [27]. These results may explain overlapping clinical symptoms in SCA3 and PD. The C-terminus of Hsc70-interacting protein (CHIP) interacts with chaperones to promote the degradation of misfolded proteins and was shown to interact with ATXN3 [28,29,30]. Further studies from Scaglione and colleagues revealed that mono-ubiquitination of CHIP by the ubiquitin-conjugating enzyme E2 W (Ube2w) induces increased ATXN3 binding to CHIP. The authors observed that ATXN3 limits the length of ubiquitin chains attached to CHIP substrates and de-ubiquitinates CHIP after the completion of substrate polyubiquitination. These findings led to the model that ATXN3 together with Ube2w regulates CHIP activity within the UPS. Interestingly, mutant ATXN3 polyQ showed increased affinity to CHIP and resulted in reduced levels of CHIP in a mouse model expression of human ATXN3 polyQ compared to the wild type [31].

Various studies found direct interaction of ATXN3 via an arginine/lysine motif (aa 117, 277–291) with valosin-containing proteins or AAA ATPase p97 (VCP/p97) and another UPS-related protein Rad23 [14,15,32].

VCP/p97 is known to target protein complexes for proteasomal degradation and for facilitating endoplasmic reticulum-associated degradation (ERAD). Together with specific ER membrane components like ER-specific E3 ligases, VCP/p97 controls the dislocation and degradation of misfolded proteins from the ER. The interaction of VCP/p97 with ATXN3 was accompanied by the reduced retro-translocation of ERAD substrates to the proteasome, indicating a regulatory role in ERAD turnover for ATXN3. Furthermore, expansion in the polyQ tract led to an accumulation of ERAD substrates compared to the wild type, suggesting a dysregulation of ERAD in SCA3 [33]. Interestingly, recent data suggest that inhibiting the interaction of VCP and ATXN3 has beneficial effects on the pathogenicity in a *Drosophila* SCA3 model [34,35].

Another protective mechanism of cells to overcome the burden of misfolded proteins is the formation of aggresomes. The misfolded proteins are actively sequestered and collected at the microtubule-organising centre (MTOC) as perinuclear inclusions and further degraded by the lysosome [36]. A direct interaction of endogenous ATXN3 with components of the aggresome forming complexes like histone deacetylase 6 (HDAC6) or dynein was observed. Also, defective aggresome formation was found in *ATXN3* siRNA knockdown cells, which was reversed by overexpression of ATXN3 protein [25].

## 6. Current Approaches for Treatment of SCA3 

Despite enormous scientific efforts, no disease-halting therapeutics for NDDs have yet been found. In general, only treatments to alleviate symptoms are available. In SCA3 and other SCAs, patients are left with general supportive management like physiotherapy, occupational therapy, and speech only [1]. Current approaches to halt NDDs concentrate on restoring neuronal plasticity in the affected brain (stem cells), removal of toxic protein aggregates (antibody therapies), and inhibition of the disease protein expression (siRNA, ASO) [37,38,39,40,41]. Recently, a CRISPR/Cas9-based targeted methylation of the PD risk gene *SNCA* has been reported in cell culture experiments, which might turn into a potential method for future therapies in a variety of NDDs [42]. 

### 6.1. ASO- and miRNA-Based Approaches for Treatment of SCA3 

For SCA3, several groups showed that allele-specific antisense oligonucleotide (ASO) approaches or AAV-based artificial microRNA gene therapy in human-derived induced pluripotent stem cell (iPSC) neurons are valid methods for the reduction in polyQ expanded and normal ATXN3 levels [43,44]. Recently, Tang et al. used an exosome-based approach to deliver the *ATXN3* targeting miRNAs, miR25, or miR181a via a non-invasive intravenous injection to overcome the often observed poor drug delivery in in vivo approaches. Exosomes were shown to penetrate the blood–brain barrier (BBB) and the authors found reduced neuronal loss, reduced neuroinflammation, and induced motor improvements in the transgenic SCA3 mouse model after 4 weeks of initial treatment [45]. 

McLoughlin and colleagues (2023) performed an *ATXN3* ASO approach to identify potential biomarkers for SCA3 and observed partially reversed neurochemical abnormalities in the brainstem and cerebellum by using magnetic resonance (MR) spectroscopy. The changes correlate with disease protein reduction and corrected motor activity in treated SCA3 mice. The results from this study confirm that ASOs have the potential to alleviate SCA3 pathology in mice models and that MR spectroscopy is a suitable non-invasive method for the detection of neurochemical changes. Finally, they found that changes in the ASO-treated SCA3 mouse model matched with the observed differences in the neurochemistry of SCA3 patients compared to healthy controls, suggesting the potential for measures as biomarkers for SCA3 [46].

### 6.2. CRISPR/CAS-Based Approaches

Originating from the bacterial and archaea defence mechanism against invading phages and plasmid transfer, the clustered regularly interspaced short palindromic repeats (CRISPR)/CRISPR-associated protein 9 (Cas) are widely used as a programmable and effective tool for genome editing [47]. 

In 2021, He et al. performed a CRISPR/Cas-mediated *ATXN3* gene correction in SCA3 patient-derived induced pluripotent stem cells and rescued the neurodegenerative conditions in these cell lines. This work proves that CRISPR/Cas is a potentially useful tool to modify patient-derived cells for subsequent stem cell transplantation to counteract neurodegeneration in SCA3 patients’ brains [48].

From the traditional CRISPR/Cas approach of inducing double-strand breaks for gene knockout or homologous repair-directed insertion of genes, further methods have been developed to modify the genome without using the nuclease activity of Cas. By mutating the coding sequence of Cas, the nuclease activity can be inactivated, which results in an inactive, so-called, dead Cas (dCas) enzyme. When the dCas sequence is fused to transcriptional activators like VP16 and p65 or repressors like Krüppel-associated box (KRAB) either a CRISPR activation or interference (CRISPRa or CRISPRi, respectively), a system for targeted modulation of gene expression is obtained. Furthermore, studies showed the possibility of modifying the epigenetic landscape by the fusion of dCas to an acetyltransferase p300 or a DNA methyltransferase DNMT3a to either increase or decrease gene expression via epigenetic modulation. These systems showed dramatically lower off-target effects and might be valuable approaches for therapeutic use. As mentioned above, studies already showed the successful, dCas9-based, methylation of the SNCA gene and its reduced expression in a cell culture model [49]. Our group performed a similar approach for a glioblastoma model cell line to increase the susceptibility against chemotherapy by reducing the expression of the O6-methylguanine DNA methyltransferase (MGMT) gene. Glioblastoma cells were transfected with the dCas9-DNMT3a/3l fusion to achieve hypermethylation of the MGMT promotor region. Hypermethylation of the MGMT promotor resulted in reduced expression of MGMT and increased the response to the chemotherapy reagent temozolomide. Furthermore, the approach seems to be highly specific for the used sgRNAs in this model as no obvious off-target effects were observed [50]. 

So far, CRISPR/Cas-based gene therapy approaches have been mainly performed in cell culture or mouse model experiments. Despite a few CRISPR-based clinical trials, several obstacles like the off-target rate, the efficiency of the respective CRISPR tool, and general limitations regarding the need for a protospacer adjacent motif (PAM) for specific gene targeting. In addition to the technical challenges, reliable drug delivery systems and the consideration of general safety issues are major points that need to be addressed in the future [49]. 

## 7. Current Clinical Trials for SCA3

Recent clinical trials for SCA3 and their progress can be found on the website of the National Ataxia Foundation (https://www.ataxia.org/pipeline/sca3/) (accessed on 15 March 2024). The following section will give a short review of the current clinical trials that already entered phase I for Human Safety Trials.

### 7.1. BHV-4157 (Troriluzole) 

BHV-4157 is a glutamine-modulating drug being developed by Biohaven Pharmaceuticals Inc. for the treatment of spinocerebellar ataxias including SCA1-3, SCA6-8 and SCA10. BHV-4157 is a prodrug, designed to overcome the limitations of riluzole, which is currently approved by the U.S. Food and Drug Administration (FDA) for amyotrophic lateral sclerosis (ALS) and Lou Gehrig’s disease. Riluzole was also shown to be safe and effective in patients with ataxia (ClinicalTrials.gov ID: NCT03408080; Study protocol). BHV-4157 is currently in a phase 3 clinical trial with 24 participants that will be completed in 2024. 

### 7.2. SLS-005 (IV Trehalose)

Trehalose is a natural disaccharide that was found to have chaperone-like activity, preventing protein aggregation and the removal of accumulated proteins by inducing autophagy. Furthermore, trehalose treatment of SCA3 cell and mouse models leads to clearance of ATXN3(polyQ), improvement of motor function, and reduction in cerebellar lesion size. A clinical phase 2 trial with 14 participants showed that trehalose was well tolerated, and no serious drug-related adverse effects were observed [51]. Currently, Seelos Therapeutics, Inc. is testing trehalose in a clinical phase 2b/3 to assess the safety and efficacy of SLS-005 in a larger cohort of 245 participants receiving intravenous infusions and is expected to be completed in 2024 (ClinicalTrials.gov ID: NCT05490563).

### 7.3. ASO (VO659) and BIIB132 

Several ASO trials for SCA3 and other ataxias are currently in the discovery and development phase. 

A first-in-human clinical trial of the ASO VO659 (Vico Therapeutics B. V.) is currently performed in a multi-centre phase 1/2a to determine its safety, tolerability, and pharmacokinetics in 65 participants suffering from genetic disorders like spinocerebellar ataxia type 1, type 3, or Huntington’s disease. Preclinical data suggests that VO659 binding to expanded CAG repeats in the mRNA of respective genes may be a valid approach for disease-modifying therapy to reduce the expression of harmful mutant proteins. The primary completion of this trial is expected in 2025 (ClinicalTrials.gov ID: NCT05822908). 

Another phase I clinical trial for SCA3 therapeutic BIIB132 (MERA study) from Biogen was stopped after careful assessment of the nonclinical safety data (https://www.ataxia.org.uk/wp-content/uploads/2023/04/Biogen-letter-to-Ataxia-UK-April-2023.pdf) (accessed on 12 March 2024).

### 7.4. Stem Cell Therapy (MSC and UC-MSC Therapy)

A phase 2 study, for efficacy and safety assessment of mesenchymal stem cells (MSC) (Stemchymal) therapy for treatment of SCA3 was completed in October 2022 by Steminent Biotherapeutics Inc. The MSCs were applied as intravenous infusions to 56 participants in a randomised, double-blind, placebo-controlled study design and the latest results are pending (ClinicalTrials.gov ID: NCT03378414).

Another clinical trial, testing the efficacy and safety of Human Umbilical Cord (UC)-MSC treatment in a random, open-label, and parallel controlled study with 45 participants started at the end of 2023. In addition to intravenous infusion, the trial will include intrathecal injection of UC-MSC in a second experimental group (ClinicalTrials.gov ID: NCT02540655).

## 8. New Targets for Treatments

Reducing the amounts or preventing the aggregation of critical disease-causing proteins by compound-based pharmacological treatments may reach the clinical phases more easily compared to the more invasive approaches such as stem cell transplantation or gene therapies.

Especially, when new properties for drugs already in clinical use are elucidated (“drug *repurposing*”), genetic targets and potential compounds affecting the expression or aggregation of respective proteins must be identified. Several ATXN3 modifier screenings have been conducted in different SCA3 disease model organisms such as *Drosophila* and *C. elegans* or human cell lines [52,53,54,55]. Two principal approaches, i.e., genetic (druggable genome) and compound modifier screenings were performed to find modulators of ATXN3 polyQ overexpression-induced toxicity by using growth and/or cell viability as a readout.

### 8.1. Genetic Modifier of ATXN3 Expression

An extensive druggable genome screen was performed in a human ATXN3 polyQ firefly luciferase reporter cell line. SiRNA knockdown of 2742 druggable genes revealed 15 genes as modifiers for ATXN3-induced toxicity, which were further validated in an SCA3 *Drosophila* model. Within the 15 genes, FBXL3, which encodes for an F-box protein that is part of the SKP1–Cullin–F-box (SCF) ubiquitin ligase complex was identified to effectively reduce toxicity. Overexpression of FXBL3 in neuronal progenitor control and SCA3 cells (NPCs) led to a reduction in endogenous wild-type and pathogenic ATXN3 levels [55].

### 8.2. Compound Modifier of ATXN3 Expression

Two compound modifier screenings to modulate ATXN3-induced toxicity have been performed in human reporter cell lines and *C. elegans,* respectively. Aripiprazole, an antipsychotic drug, was identified in an unbiased primary screening of 1250 FDA-approved drugs in an SCA3 cell model. Further validation in *Drosophila* and mice SCA3 models revealed increased longevity in flies and a reduction in aggregated ATXN3 species in flies and mice brains after aripiprazole treatment [54]. Recently, a screening of 3942 compounds including many FDA-approved substances has been performed in an ATXN3 polyQ overexpressing *C. elegans* model. Five compounds were identified to rescue the motor-deficient phenotype, protecting against neurodegeneration and increasing the life span in transgenic worms. When investigating a potential mechanism behind the beneficial effects of these molecules, the authors found that three out of five molecules, namely chenodiol (CHEN), fenbufen (FEN), and sulfaphenazole (SULFA) act as modulators for the transcription factor EB (TFEB/HLH-30), a key factor in autophagy pathways. In TFEB/HLH-30 knockout worms, the beneficial effects were not observed indicating a critical role of this TF in rescuing ATXN3 polyQ-induced phenotype. On the other hand, overexpression of TFEB/HLH-30 alleviates the motility defect in SCA3 worms. From the three compounds modulating TFEB/HLH-30, FEN is already clinically used as a non-steroidal and anti-inflammatory drug in AD to prevent inflammatory processes observed in patients and thus may have the potential as a new treatment option for SCA3 patients [53]. In a recent in silico analysis of phytochemicals, the flavonoid chamanetin was predicted to be highly active against ATXN3 but in vitro analysis is pending [56] (summarised in Table 1).

Finding novel therapeutic targets and compounds affecting the expression levels of disease-causing proteins in NDDs is the prerequisite for the development of disease-modifying or halting therapies. High throughput screenings (HTS) as described above are suitable approaches to identify yet unknown genetic or compound modifier candidates for therapies of NDDs. Almost all of the studies consider the pathologically observed hallmark of protein aggregation and used disease protein overexpression-induced toxicity models for genetic or compound modifier libraries. Thus, any potential gene or compound candidates modulating the epigenetic, transcriptional, and posttranscriptional regulation of misfolding proteins might be missed. 

**Figure 3:**
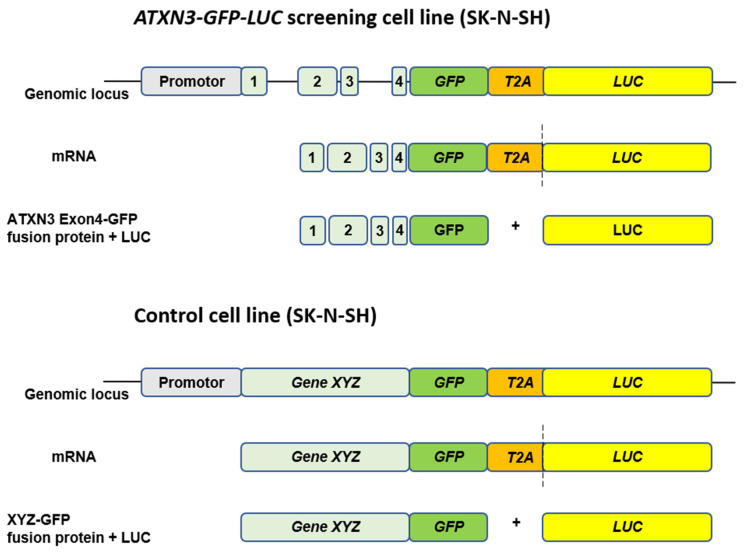
Reporter cell line-based compound screening for modulators of ATXN3 expression modulating drugs. Schematic overview of CRISPR/Cas9 genome editing-based generation of *ATXN3-GFP-LUC* screening and control cell lines. The integration of a *GFP*-*T2A-LUC* cassette under the endogenous promotor of *ATXN3* enabled a fast and efficient readout for modulators of transcription levels. Translation of the mRNA results in an ATXN3-Exon4-GFP fusion protein and a functional LUC enzyme that are separated by a ribosomal skipping event mediated through the T2A sequence. The control cell line served as a specificity control to exclude unspecific modulators.

We had thus chosen another HTS approach for the identification of endogenous and transcriptional modulators of *ATXN3* expression via human luciferase reporter cell lines (Figure 3). The aim of the studies was to find compounds which significantly modulate the expression of endogenous ataxin-3 and to identify new epigenetic, transcriptional, or posttranscriptional mechanisms for these changes in human cell line-based screenings [58].

In brief, we screened a library containing 2640 bioactive and FDA-approved drugs but identified no unequivocal transcriptional inhibitors for *ATXN3*. Statins were identified as potential activators of *ATXN3* expression in the primary luciferase assay-based HTS. The screening library contains eight statins from which atorvastatin, mevastatin, Fluvastatin, and simvastatin alter luciferase signals in the screening cell line. Simvastatin showed the most consistent effect and was confirmed to raise ataxin-3 mRNA and protein levels in non-modified SK-N-SH wildtype cells. Statins represent a potent compound class halting the conversion of 3-hydroxy-3-methylglutaryl coenzyme A to mevalonate by inhibiting HMG-CoA reductase (HMGCR), the key enzyme in cholesterol homeostasis [59]. 

The inhibition of HMGCR leads to reduced sterol levels in the cell and activates SREBPs to initiate the transcription of genes involved in cholesterol homeostasis. Increased ATXN3 levels are likely mediated through direct binding of the sterol regulatory element binding protein 1 (SREBP1) TF to the *ATXN3* promotor as shown in ChIP-qPCR assays. The human and murine *ATXN3* promotor contains binding motifs for SREBP1. Overexpression of active human SREBP1a in murine Neuro 2a cells (N2a) led to an increase in endogenous ATXN3 protein suggesting a similar SREBP1-dependent regulation of murine and human ATXN3 levels [58]. Based on our data, a yet unappreciated role of ATXN3 in the regulation of cholesterol synthesis is likely.

Inhibition of HMGCR results in cholesterol deprivation and induces the activation of SREBPs. Three SREBPs have been identified and found to function as classic basic helix–loop–helix leucine finger (bHLH zip) transcription factors, which are involved in cholesterol (SREBP1a/SREBP2) and fatty acid (SREBP1a/SREBP1c) homeostasis. SREBP1a/c are transcribed from one gene (*SREBP1*) and differ in the N-terminal transactivation domain whereas SREBP2 is encoded by the *SREBP2* gene. SREBPs are located in the endoplasmic reticulum membrane and directly bind to SREBP cleavage-activating protein (SCAP), which senses cholesterol levels in the cell. Under low cholesterol levels, SCAP escorts SREBP via COPII vesicles to the Golgi where site-1 and site-2 proteases release the active (mSREBP) TF for nuclear translocation and transcriptional activation. At normal or high cholesterol levels, SCAP retains SREBP in the ER membrane by direct interaction with insulin-induced genes 1 and 2 (INSIG1, INSIG2) [60]. 

## 9. Cholesterol in SCA3

Only a few studies linked altered cholesterol homeostasis to SCA3. Toonen and colleagues (2018) found transcriptional changes in genes related to cholesterol biosynthesis pathways in the brains of an SCA3 mouse model [61]. Another study reported that cholesterol 24-hydroxylase (CYP46A1), which converts cholesterol into 24-hydroxylcholesterol (24-OHC) enabling cholesterol turnover and efflux via the BBB into circulation, was reduced in cerebellar extracts of SCA3 patients and in an SCA3 mouse model. The authors showed that overexpression of CYP46A1 in SCA3 mice led to reduced accumulation of ATXN3polyQ and neuroprotection, whereas knockout of CYP46A1 impairs cholesterol metabolism and leads to severe neurodegeneration [62]. 

CYP46A1 expression was also found to be reduced in other NDDs including AD and HD in patient-derived post mortem brain samples and mouse models suggesting a more general role of this enzyme in brain plasticity and protection against neurodegeneration. Also, overexpression of CYP46A1 was shown to have beneficial effects in neuroprotection, implementing that cholesterol turnover and an efflux rate of 24-OHC are probably critical in NDDs [63]. 

Despite the above-mentioned implications of cholesterol synthesis or turnover in SCA3, none of the studies has yet identified a potential function of ATXN3 in cholesterol homeostasis, although *ATXN3* is likely a SREBP1 regulated gene and may play a (yet) unknown role in the SREBP dependent regulation of cholesterol homeostasis [58]. HMGCR, the key enzyme in cholesterol homeostasis, is an ERAD-dependent substrate and is degraded under high cholesterol conditions. Ubiquitination of HMGCR is initiated via the binding of INSIG, which in turn is associated with VCP/p97, the ubiquitin-conjugating enzyme E2 (Ubc7) and glycoprotein 78 E3 ubiquitin ligase (gp78) [64]. 

**Figure 4:**
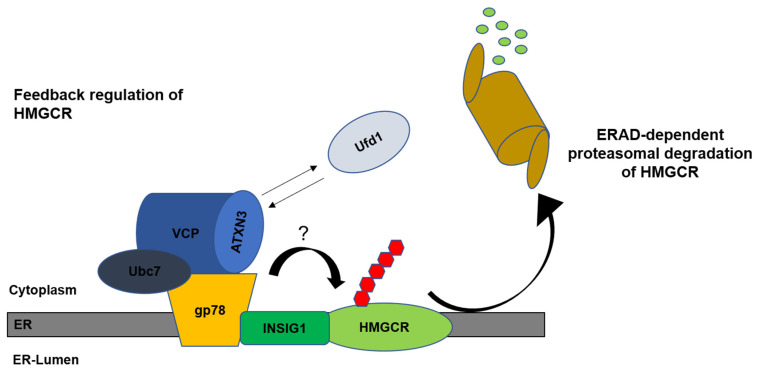
Putative role of ATXN3 in the feedback regulation of HMGCR. HMGCR is undergoing ERAD-dependent proteasomal degradation after restoration of sterol or cholesterol levels. ATXN3 may attenuate the HMGCR flow through ERAD by interacting with VCP. Direct interaction of ATXN3 and VCP inhibits binding of VCP and Ufd1 and retro-translocation of ERAD substrates from the ER [33]. Modified from [65]. ER, endoplasmic reticulum; VCP, valosin-containing protein; Ubc7, ubiquitin-conjugating enzyme E2 7; gp78, glycoprotein78 E3 ubiquitin ligase; INSIG1, insulin-induced gene 1; HMGCR, 3-hydroxy-3-methylglutaryl coenzyme A reductase; ATXN3, ataxin-3; Ufd1, ubiquitin recognition factor in ER associated degradation 1.

Earlier studies reported that ATXN3 directly interacts with VCP/p97 and plays a potential role in regulating the flow and turnover within the ERAD pathway. Thus, it is tempting to hypothesise that ATXN3 could also be involved in HMGCR stability (Figure 4). Since the expansion of the polyQ tract of ATXN3 affects the binding to its substrates, HMGCR turnover in SCA3 patients might be altered, leading to impaired cholesterol homeostasis and possibly neurodegeneration. However, these findings have only limited value for the assessment of whether statins should be omitted in the pharmacological treatment of SCA3 patients, as it is difficult to predict whether the low statin-induced expression changes in ATXN3 might evolve critically in the long run.

## Figures and Tables

**Table 1:** Summary of screening approaches to identify modifiers for ATXN3. Studies that performed genetic or compound modifier screenings to identify modulators of ATXN3 levels by using different approaches, readouts, and cell models or SCA3 model organisms.

Scheme	Readout	Compound/Gene	Action	Organism	Author, Year
Druggable genome screen	Overexpression induced toxicity	FBXL3 (and others)	SKP1–Cullin–F-box (SCF) ubiquitin ligase complex Overexpression FBXL3 reduced ATXN3 level	HEK 293 reporter cells *Drosophila*	Ashraf et al., 2020 [55]
RNAi screen	Overexpression induced toxicity	Several genes	Involved in protein turnover/quality control; nuclear import/export and mRNA transport, editing, translation	*Drosophila*	Vossfeldt et al., 2012 [52]
RNAi TF screen	Overexpression induced toxicity	FKH-2/FOXG1	Ortholog of human forkhead box G1 (FOXG1), associated rare disorder with impaired development and structural brain abnormalities Reduced overexpression-induced phenotypes	*C. elegans*	Fardghassemi and Parker 2021 [57]
Drug screen	Overexpression induced toxicity	Aripiprazole	Increased longevity in flies, reduction in aggregated ATXN3 species in flies and mice brains	HEK 293 reporter cells *Drosophila* Mice	Costa et al., 2016 [54]
Drug screen	Overexpression induced toxicity	Chenodiol, fenbufen, and sulfaphenazole	Modulators of TFEB/HLH-30 TF Reduced overexpression-induced phenotypes	*C. elegans*	Fardghassemi et al., 2021 [53]
Drug screen	Endogenous expression of ATXN3 LUC signal (primary screen)	Simvastatin	Activator of ATXN3 expression	Endogenous SK-N-SH *ATXN3-GFP-LUC* fusion reporter cell line, SK-N-SH wild type and SCA3 patient-derived NSC	Stahl et al., 2023 [58]
In-silico modelling	------	Flavonoid chamanetin	Highly active against ATXN3	In-vitro/vivo studies are pending	Naveed et al., 2024 [56]
